# Wildlife laws monitoring as an adaptive management tool in protected area management in Ghana: a case of Kakum Conservation Area

**DOI:** 10.1186/s40064-016-3129-x

**Published:** 2016-08-30

**Authors:** Edward Debrah Wiafe

**Affiliations:** Department of Environmental and Natural Resources Management, Presbyterian University College, Ghana, P.O. Box 393, Akropong–Akuapem, Ghana

## Abstract

**Introduction:**

The wildlife laws of Ghana alienated the rural communities from forests and material well-being depended upon for their livelihood and this manifests itself in the progressive conflict between the park patrol staff and poachers from the fringes of the protected areas.

**Case description:**

The main aim of this study was to determine the impact of quantification of patrol efforts on indicators of illegal hunting activities that occur in rainforest protected areas, as a result of monitoring patrol operations and modifying the original plan. The specific objectives were to determine the optimal patrol efforts necessary to reduce illegal wildlife use to minimal; and the influence of the rainfall and seasonal activities on illegal wildlife use.

**Discussion and Evaluation:**

The results indicated that as the patrol efforts increased the encounter with illegal wildlife use also increased until a certain point that the encounter rates started decreasing. Neither rainfall nor seasonal activities influenced the illegal activities and the patrol efforts. The protection staff of rainforest protected areas would work effectively to bring down illegal wildlife off-take to the barest minimum if monitored, quantified and provide feed-back.

**Conclusions:**

Illegal wildlife off-take can also be reduced by the protection staff if the original plans are made flexible to be adjusted. Recommendations for further studies have been made.

## Background

The decline of forests and wildlife resources in the tropics require much attention and the efforts to arrest these declines have become part of the government policies and laws. Since the beginning of the twentieth century, land has been demarcated for conservation with little or no concern for the impact of these on the livelihood of the inhabitants of rural communities in Africa. Consequently, these communities were alienated from the resources upon which their material well-being depends. Instead of re-investment of the revenues derived from wildlife back into the area, they were channelled into the Government’s central treasury. As a result, many local hunters and gatherers operate clandestinely for personal gain and it also compels many people into the illegal, subversive economy (Jachmann 1998).

The main objective of the protected area management is to protect the integrity of the ecosystems with their biodiversity and to conserve the representative biological samples of all ecological regions. However, the majority of protected areas are islands surrounded by settlements and agricultural farms (Struhsaker 1997). Brashares et al. (2004) are of the view that though fragmentation resulting into small reserves cause decline in biodiversity; bushmeat hunting has been the main activity or reason for significant decline in wildlife populations.

Hunting can trigger the alteration of the overall function, structure and composition of the ecosystem. Despite the straightforward effect of hunting activities on targeted species, it may also have cascading effects on the entire biological diversity (Wright 2003). In spite of these, hunting has been enjoyed by Africans since their existence on the continent and an estimated 74 % of sub-Saharan African protein is derived from bushmeat (Asibey 1974). Furthermore, 70 % of Ghanaians still eat bushmeat and 90 % have indicated their willingness to eat it upon its availability (African Center for Economic Transformation [ACET] 2014).

To achieve the needs of wildlife users without compromising the conservation goals, several measures have been instituted. Among these are the protected area management system and its associated laws to regulate wildlife utilization. These modern concepts arrived in Africa with colonial rule (Jachmann 1998), hitherto, the consumptive use of wildlife was enshrined in local traditions and beliefs (Appiah-Opoku 2007). The new conservation laws deprived the local people of their hunting rights in the protected areas forgetting that different cultural backgrounds and values result in differences in wildlife appreciation. As a result, the hunters living around protected areas operated surreptitiously for short term economic gain, disrespecting most of the conservation laws (Jachmann 1998).

The continuous struggling between wildlife law enforcers and hunters indicates some discrepancies in enforcing conservation laws. In Ghana, the importance of properly planned and executed law enforcement programmes has been underrated and most of them were conducted on an ad-hoc basis. Adaptive management is a relatively new concept that incorporates research into conservation action. Specifically, the concept integrates design, management and monitoring to systematically test assumptions in order to adapt and learn. Adaptive management can further be viewed as the process of hypothesizing how ecosystems management should have worked with people, monitoring results, comparing them with expectations and modifying management decisions to better achieve conservation objectives through improved understanding of ecological processes (Lancia et al. 1996; Salafsky et al. 2001). Adaptive management, a modern concept of testing a management plan, monitoring how it works, and using the result to adjust the original plan was adopted and tested in the protected area law enforcement program from January 2005 to December, 2009 in Kakum Conservation Area (KCA), Ghana. In mid-2004, an inexpensive patrol based monitoring system based on East African models (Bell 1985; Bell et al. 1992; Jachmann 1998) was initiated in KCA. The study had dual objectives: firstly, to document the impact of quantifying patrol efforts on incidence of illegal wildlife use and secondly to examine the relationships between factors that can influence hunting incidence and patrol performance.

## Methods

### The study area

**Fig. 1:**
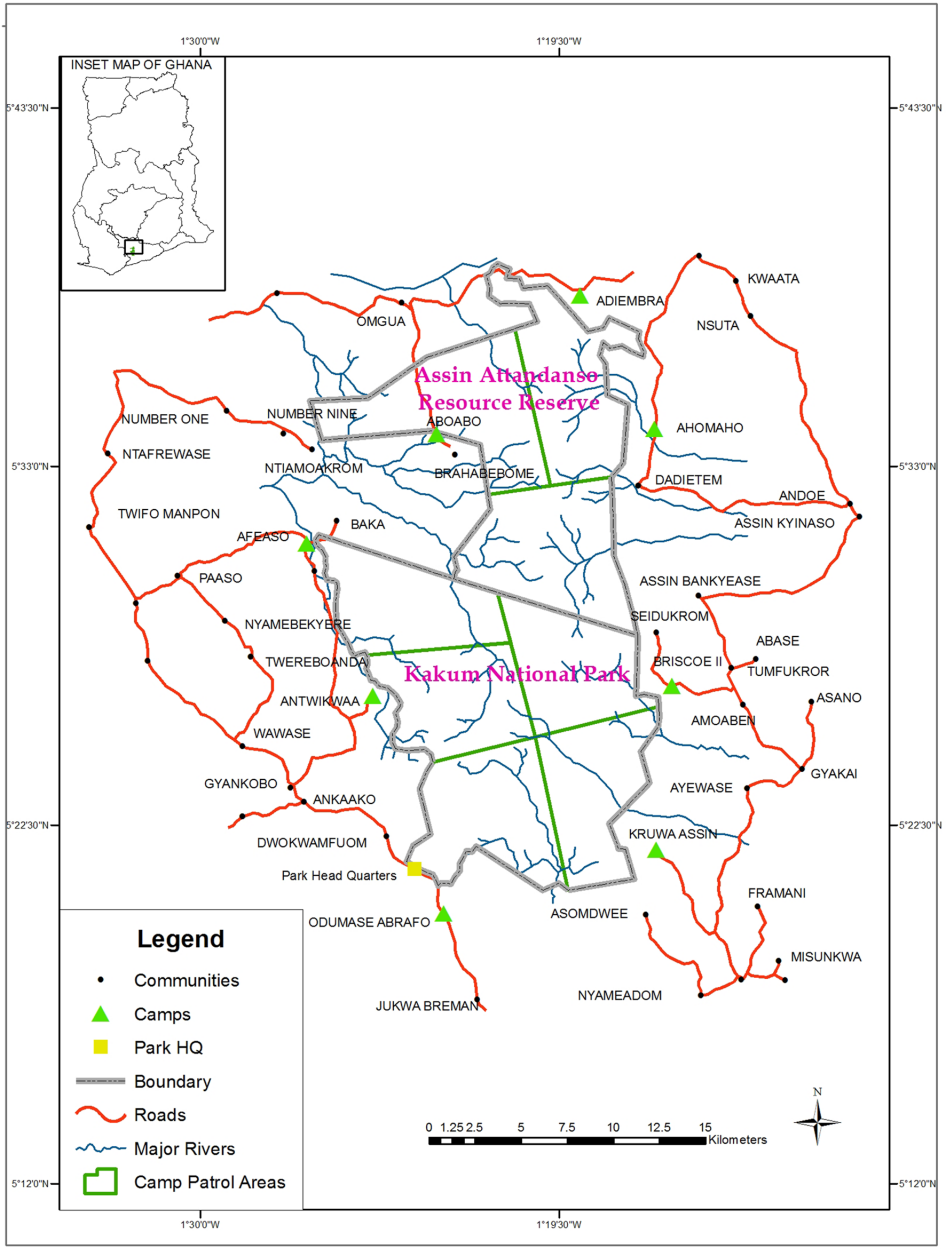
Map of Kakum Conservation Area showing the distribution of communities, patrol area and camps

The study took place in Kakum Conservation Area (KCA) located at longitude 1°30′W and 1°51′W and latitude 5°20′N and 5°40′N, made up of the 210 km^2^ Kakum National Park (KNP) and its twin 150 km^2^ Assin Attandanso Resource Reserve (AARR). The regulations of national parks and resource reserves prohibit entry, collection and use of any resource in the reserves without a written permit. It spans the Twifu Hemang Lower Denkyira, Assin (North and South) and Abura–Asebu–Kwamangkese districts of the Central Region of Ghana. The Kakum Forest and Assin Attandanso Forests were legally regazetted as a national park and resource reserve respectively in 1991 under the wildlife reserves regulations (L.I 1525) under the administrative jurisdiction of the Wildlife Division of the Forestry Commission (Wildlife Department 1996). The area was initially placed under timber production by the Forestry Department until 1989 when its management was transferred to the Wildlife Division because of the change in management status. About 52 communities are scattered around (outside) the conservation area ( Fig. 1).

### Data collection

The data on illegal hunting activities were collected between 2005 and 2009. Eight (8) camps have been established in eight communities at peripheries of the conservation area. Each camp is manned by at least five (5) well-trained wildlife rangers led by the senior-most of them. The main purposes of the camps are to house the rangers to execute their duties and to deter poaching or arrest poachers.

The conservation area uses conventional law enforcement in the form of foot patrols that frequently range out from each of the camps as well as from the headquarters. Patrol routes can be viewed as transects with unfixed width used to collect information on indicators of illegal wildlife use. Standardized data sheets were used to keep records of the numbers of staff on patrol; the exact duration; the area travelled as well as types, quantity and locations of illegal activity encountered. Because patrol movements should be unpredictable by nature, the officers were trained to randomize patrol movements as much as practically feasible to optimize impact on illegal off-take and to enable statistical inference from data. The patrol time used here was the effective time spent actively in pursuit of illegal activities as described by Jachmann (1998, 2008). Patrols took place either by day, at night time or both, respectively as day patrol, night patrol or long patrol. Patrol efforts were measured in terms of man-days per month or year and related to the trends of indicators of hunting activities during the same period. Patrol man-days are equivalent to number of staff on patrol multiplied by the number of days or hours patrolled (where 8 h patrol period is equivalent to 1 day). Indicators of hunting activities were categorized according to those offences which directly relate to wildlife killing which include poachers arrested, poachers observed, firearms confiscated, spent cartridges found, skins confiscated, gunshots heard, poacher’s camps found, animals found killed, wire snares recovered and carbide ashes found (Wiafe and Amoah 2012). Monthly rainfall data was obtained from the Ghana Meteorological Service recordings at Asuansi Weather Station, the closest weather station to KCA (about 3 km away from the park).

### Patrol operations and management

During patrol operations, leaders of the patrol teams discussed the patrol route with the team members and take records of the patrol including coordinates of the starting point, names of the team members and the time of departure. In course of the patrol the team may decide to change the direction (north, south, east, west, etc.); patrol movement style or decide to lay armed bush depending on the observation made.

Monthly meetings were held at the park headquarters to discuss collated patrol report. At these meetings, based on the observations made and lessons learnt, the next strategy would be adopted. For example either a particular camp switches from day patrol to night or embarks on long patrol or a combination of two or more teams for re-enforcement patrol. In addition, patrol movement style can also be changed from a ‘single file’ to an ‘arrow head’, depending on the lessons learnt from the previous patrol.

### Data analysis

A Catch per Effort index (C/E) was used to measure the levels of indicators of hunting activities per period (Jachmann 1998). Catch refers to the total number of monthly encounters with indicators of hunting activities and the effort is the total number of effective patrol man-days per month (Jachmann 2008; Bell 1985). In this study the catch can be referred to as encounters per man-day per month. Kilometric Index of Abundance (KIA), the ratio of illegal activities encountered to distance in kilometers (km) walked per month, was used as another measure of encounter rates (Groupe 1991). Kruskal–Wallis tests were conducted to evaluate the differences of the variables between more than 2 years or events and Mann–Whitney U tests were used to evaluate the difference between two events. Spearman’s rank correlation was used to find the relationship between indices of hunting/illegal activities and rainfall records.

## Results and discussions

### Trends of patrol efforts

The annual average man-day per month in 2005, 2006, 2007, 2008 and 2009 were as follows: 211.8 (SD = 50.0), 266.8 (SD = 115.0), 289.9 (SD = 31.7), 415.8 (69.4) and 434.5 (SD = 72.8) respectively. The average monthly man-days of patrol effort increased significantly from the first year, 2005, to the fifth year, 2009 i.e., from ca. 212 (SD = 50.0) to 435 (72.8) in 2009 (H = 36.48, p < 0.05) Patrol teams walked a monthly average of 193.2 (SD = 60.5) km in 2005; 308.2 (SD = 113.3) in 2006; 306.7 (SD = 41.7) in 2007; 407.3 (SD = 75.2) and 663.2 (SD = 77.9) in 2008 and 2009 respectively. Monthly distances walked per year vary significantly across years (H = 44.04, p < 0.001) and aside from the distances covered in 2006 and 2007 that did not differ, all the distances differed significantly. On the whole, across the 60 months of patrols, the number of man-days per month was positively correlated with number of kilometers walked in those months (rs = 0.876, p < 0.001). The higher the number of staff on patrol the larger the area covered. Influence of man-days on distance coverage cannot be overemphasized, as the number of patrol staff increases they are able to patrol for a long period and cover longer distances.

### Determining factors of patrol efforts

Man-days by month across all the years did not vary (H = 4.26, *p* = 0.96) indicating that monthly conditions did not influence patrol man-days. For instance monthly salaries for staff and supply of logistics were the same across month and did not influence patrols.

Most of the time, in Ghana, the wildlife protection staff working in rainforest protected area cultivate farms to supplement their income during their off days (Wiafe and Amoah 2012). It had therefore been speculated that these farming activities disrupt patrol efforts and therefore poaching activities rise up during the farming months. However, farming activities neither emerged as a factor disrupting the distances covered by the patrol staff (U = 448.5, *p* = 0.98) nor man-days used i.e., number of staff and hours patrolled (U = 431.5, *p* = 0.79). The encounter rates of the illegal activities during farm cultivation period i.e., between February to April did not vary widely from non-farm cultivation period (Tables 1, 2).

**Table 1:** Catch per effort indices (indicators of hunting activities/distance covered) in terms of distances of indicators of hunting activities

Month	2005	2006	2007	2008	2009	Average
January	0.62	0.1	0.24	0.34	0.11	0.28
February	0.38	0.24	0.11	0.37	0.1	0.24
March	0.15	0.19	0.2	0.21	0.08	0.17
April	0.26	0.22	0.42	0.19	0.16	0.25
May	0.59	0.17	0.32	0.4	0.11	0.32
June	0.22	0.29	0.47	0.17	0.06	0.24
July	0.56	0.2	0.5	0.28	0.08	0.32
August	1.10	0.39	0.45	0.52	0.11	0.51
September	0.45	0.22	0.3	0.32	0.08	0.27
October	0.29	0.17	0.33	0.25	0.13	0.23
November	0.29	0.25	0.37	0.24	0.15	0.26
December	0.49	0.18	0.57	0.23	0.18	0.33
Average	0.45	0.22	0.36	0.29	0.11

**Table 2:** Catch per effort indices (indicators of hunting activities/distance covered) in terms of man-days of indicators of hunting activities

Month	2005	2006	2007	2008	2009	Average
January	0.55	0.11	0.25	0.5	0.18	0.32
February	0.31	0.26	0.13	0.32	0.14	0.23
March	0.12	0.22	0.26	0.25	0.11	0.19
April	0.21	0.24	0.45	0.15	0.21	0.25
May	0.61	0.2	0.34	0.22	0.17	0.31
June	0.21	0.3	0.51	0.16	0.1	0.26
July	0.52	0.22	0.54	0.27	0.11	0.33
August	0.94	0.67	0.45	0.49	0.17	0.54
September	0.44	0.26	0.3	0.31	0.13	0.29
October	0.21	0.2	0.36	0.26	0.21	0.25
November	0.3	0.35	0.41	0.27	0.24	0.31
December	0.51	0.24	0.48	0.26	0.27	0.35
Average	0.41	0.27	0.37	0.29	0.17	

Another assumption had been that precipitation disrupts patrol efforts in terms of the number of staff willing to work. Similarly, precipitation was also found to be not significantly correlated with the man-days used [Spearman (rs) = 31,990, *p* = 0.23] or the distances patrolled [Spearman (rs) = 33,976, p = 0.67]. This may imply that the protection staffs were still working to reduce illegal hunting activities even under uncomfortable weather conditions which the poachers might have taken advantage to operate. It must be noted that some of the principles of adaptive management (Salafsky et al. 2001) were in consideration.

### Trends of hunting indicators

**Fig. 2:**
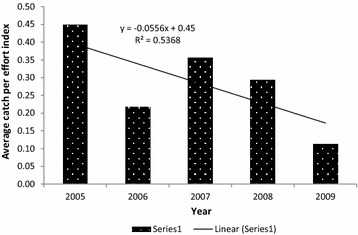
Average Kilometric Index of Abundance (observation of indicators of illegal hunting activities per distance covered [km]) per year

**Fig. 3:**
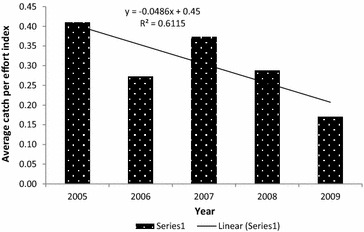
Average catch per effort indices (observation of indicators of illegal hunting activities per man-days used per month) per year

The encounter rates of all activities indicating killing and capturing of wild animals were relatively high at the beginning of the project in 2005, in terms of both man-days used and distance covered. Figures 2 and 3 indicate average annual trends of encounter rates both in terms of distance patrolled and man-days used for patrol in the various years respectively. In both cases, downward trends were observed as the years advanced and that can be attributed to experiences obtained (learning and adopting).

**Fig. 4:**
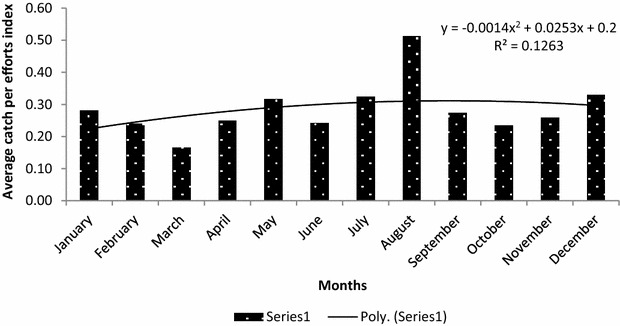
Trends of encounter rates (KIA) of indicators of illegal hunting activities in terms of distance covered in km

**Fig. 5:**
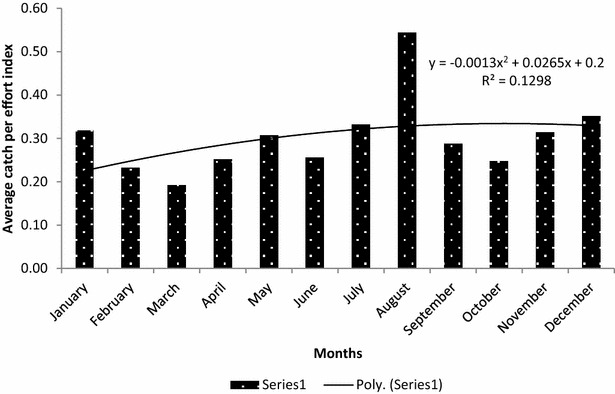
Trends of encounter rates (CE) of indicators of illegal hunting activities in terms of man-days used

Furthermore, trends of average encounter rates of illegal hunting activities on monthly basis over the 60 month period have respectively been shown in Figs. 4 and 5 for both distance covered and man-days used. At the end of the every month, the staff met, discussed their observations and where necessary, modified their patrol strategy to suit the conditions that would lead to either deter poachers or arrest them. The inconsistency in trends of encounter rates can be attributed to monthly socio-economic and legal environment that may be operating in a particular month in the country.

**Fig. 6:**
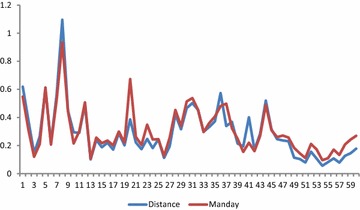
Trend of encounter rates of illegal activities in terms of man-days and distance

The patrol staffs were at liberty to change their patrol strategies and style, at any time, in order to achieve patrol objectives but it depended on what the patrol staffs have experienced. Reference to Fig. 6, some oscillation remained but the encounter rates did not increase beyond a certain threshold (1.59). At the beginning of the project, an arbitrary encounter rate of 0.05 was set as the target which illegal hunting activities would be reduced to, in terms of distance covered and man-days used. However, the minimum encounter rate did no go below 0.09 over the 5 year period (Tables 1, 2; Fig. 6).

Management plans strictly adhered to cannot function in the present day natural resources management because of the following factors enumerated by Salafsky et al. (2001) as follows: (1) The complex systems where conservation projects take place; (2) the constantly and unpredicted changing world; (3) the changing and adapting behavior of the competitors; (4) the requirement of immediate action; (5) no complete information exists and (6) managers can learn and improve. It is therefore, apparent that from January 2005 to December, 2009 protection staffs at KCA have been learning and improve upon their operations.

Though there were many factors (such as financial, events in the year, legal and political) that had the tendency to contribute to illegal hunting and efforts to bring it down, the study only hypothesized and tested rainfall and farming seasons. In addition, internal factors such as leadership styles, logistics supply, and training could influence on field patrolling and other related operations this study did not consider it.

## Conclusions

Wildlife managers should know whether or not they are doing an effective job of managing natural resources and the kind of decision they make should result in a proper accountability to the public and to conservationists.

In 1990, the management objectives of the forest were changed from production of timber to protection and conservation objectives. However, the reporting of events was not quantified but was qualitatively described. This made monitoring of wildlife, factors affecting it and staff performance difficult and on an ad-hoc basis. Therefore, it became necessary to introduce a new concept of staff deployment to patrol and report events and other factors affecting the patrol operations.

Form the result, it could be deduced that the trend of the encounter rates fluctuates between months. It shows that the result of a particular month and lessons learnt were used to influence the activity of the patrol (in terms of identifying secrete hunting) of the preceding month.

Since patrol members were flexible in implementation of management plans and strategies they contributed to reducing the encounter rates to the barest minimum. In spite of the financial constraints, with often no more a trickle of operational funds flowing to the field, the patrol staffs have done a tremendous good job under difficult circumstance and if it continues illegal wildlife off-take would be reduced drastically.

It is therefore recommended that the system be adopted and implemented as a standard mode of operation and reporting in all protected areas suffering from illegal hunting. Further studies on influence of external and internal factors on illegal off-take of forest and wildlife resources and efforts of maintaining sustainable utilization is also recommended.
